# Prey density affects predator foraging strategy in an Antarctic ecosystem

**DOI:** 10.1002/ece3.5899

**Published:** 2019-12-13

**Authors:** Karl M. Busdieker, Samantha C. Patrick, Alice M. Trevail, Sébastien Descamps

**Affiliations:** ^1^ School of Environmental Sciences University of Liverpool Liverpool UK; ^2^ Norwegian Polar Institute Fram Centre Tromsø Norway

**Keywords:** Antarctic petrel, habitat selection, prey defense, prey density, south polar skua

## Abstract

Studying the effects of prey distribution on predator behavior is complex in systems where there are multiple prey species. The role of prey density in predator behavior is rarely studied in closed ecosystems of one predator species and one prey species, despite these being an ideal opportunity to test these hypotheses. In this study, we investigate the effect of prey density on the foraging behavior of a predatory species in an isolated Antarctic ecosystem of effectively a single predatory species and a single prey species. We use resource selection models to compare prey density in areas utilized by predators (obtained from fine‐scale GPS telemetry data) to prey density at randomly generated points (pseudoabsences) throughout the available area. We demonstrate that prey density of breeding Antarctic petrels (*Thalassoica antarctica*) is negatively associated with the probability of habitat use in its only predator, the south polar skua (*Catharacta maccormicki*). Skuas are less likely to utilize habitats with higher petrel densities, reducing predation in these areas, but these effects are present during chick rearing only and not during incubation. We suggest that this might be caused by successful group defense strategies employed by petrel chicks, primarily spitting oil at predators.

## Graphical abstract

We used habitat selection models to analyse the effect of prey density on predator behavior in a uniquely straightforward system of one predator species and one prey species. We found that probability of habitat use by south polar skuas is negatively associated with antarctic petrel density and that this effect varies depending on whether predator or prey is incubating eggs or brooding chicks. We suggest that this effect is a result of successful group defense strategies employed by petrels.

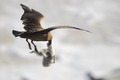

## 1 INTRODUCTION

Nest predation is one of the main causes of reproductive failure in birds (Martin, 1993; Ricklefs, 1969), and numerous strategies have evolved among bird species to reduce this risk. Coloniality has been proposed to represent one of these strategies (Lack, 1968; Rolland, Danchin, & de Fraipont, 1998) by increasing group vigilance or defense (Elgar, 1989), thus reducing vulnerability to predation. In colonial species, patches of high prey density simultaneously offer high potential resource gain to predators but also represent areas where group defense will be the most effective (Sih, 1984; Varela, Danchin, & Wagner, 2007) and costs of foraging may potentially be higher. This may be especially true in systems where prey can actively defend against predation. In such systems, it remains unclear whether predators should avoid areas of high prey density because the associated foraging costs are too high, or alternatively, if they should target them because they offer the highest energetic benefits.

The role of prey density in driving predator foraging behavior is difficult to assess in many systems, where predators are able to switch prey (Abraham & Sydeman, 2006) and prey may have to defend against multiple predators (Sih, Englund, & Wooster, 1998). Therefore, to comprehensively test how predators adapt their behavior to the distribution of prey, a system with a single predator and prey species is ideal. On mainland Antarctica, there are inland seabird colonies characterized by a single prey species, the Antarctic petrel, *Thalassoica antarctica*, and a single predator, the south polar skua, *Catharacta maccormicki*, (Brooke, Keith, & Røv, 1999) which offer the ideal opportunity to study such predator–prey interactions. Antarctic petrels, much like many seabird species, are a colonial organism, and additionally adults and chicks are able to deter attacks from predators by ejecting stomach oil that severely compromises south polar skua plumage and survival (Warham, 1977). Chicks in particular benefit from group vigilance (Dimond & Lazarus, 1974; Elgar, 1989; Seddon & van Heezik, 1993), and this is likely especially true once they are no longer continuously guarded by a parent on the nest.

Previous studies have analyzed the diet and feeding habits of the south polar skua in detail (Young, (1963a); Malzof and Quintana (2008) and Reinhardt, Hahn, Peter, and Wemhoff (1998)). All of these studies use sites in which skuas have access to multiple prey sources (e.g., several seabird species and marine resources such as fish; Malzof & Quintana, 2008; Mund & Miller, 1995; Pietz, 1987) and are able to switch between these sources when necessary. Here, we use data on the nest density of Antarctic petrels and spatial behavior of south polar skuas from Svarthamaren, Dronning Maud Land, Antarctica, to examine the importance of petrel nesting density in driving skua foraging behavior. The colony site at Svarthamaren is 1,600 m above sea level and is located approximately 200 km inland (Mehlum, Gjessing, Haftorn, & Bech, 1988), preventing skuas from feeding at sea as they otherwise might when terrestrial prey is scarce (Carneiro, Manica, Trivelpiece, & Phillips, 2015). This exclusively inland feeding is corroborated by the findings of Brooke et al. (1999) in the same colony. Therefore, our study, in a system where the option of switching to an alternative food source in not available, provides an ideal platform from which to assess the importance of prey density in predator foraging behavior.

The foraging behavior of seabirds may change during the breeding season (Navarro et al., 2009) and in particular depends on the predator and/or prey breeding status. Indeed, the energetic demands are not the same during egg incubation and chick rearing which affects the foraging behavior of breeding seabirds (Ito, Takahashi, Kokubun, Kitaysky, & Watanuki, 2010; Weimerskirch, Salamolard, Sarrazin, & Jouventin, 1993). Additionally, the costs and benefits associated with a specific prey may also vary with the prey breeding status. Consequently, the role played by the prey density may vary within a breeding season and be dependent on both the prey and the predator breeding phenology. Foraging in seabirds may also be dependent on sex, with males and females exhibiting different foraging behaviors and diet during the breeding season (Forero et al., 2002; Lewis et al., 2002; Navarro et al., 2009). Considering the potential importance of the predator sex and breeding status, and the prey breeding status, we tested the following hypotheses: petrel density drives skua foraging activity, and thus habitat use (a); its effect depends on both the skua and petrel phenology (b); and may be sex‐dependent (c).

## 2 METHODS

This study was conducted during the 2012/2013 breeding season at Svarthamaren (Dronning Maud Land, Antarctica 71°53′S, 5°10′E) in a seabird colony dominated by Antarctic petrels (approx. 200,000 pairs of Antarctic petrels, 1,000 pairs of snow petrels and 150 pairs of south polar skuas) (Descamps et al., 2016; Mehlum et al., 1988; Varpe & Tveraa, 2005). Svarthamaren mountain is dominated by scree slopes, and above these slopes the rock is almost vertical. Two major “amphitheaters” on the NE side screes are home to the petrel breeding colony, which presents pronounced gradients in Antarctic petrel density, with skuas nesting beneath on a narrow strip of flat ground (Mehlum et al., 1988).

**Figure 1:**
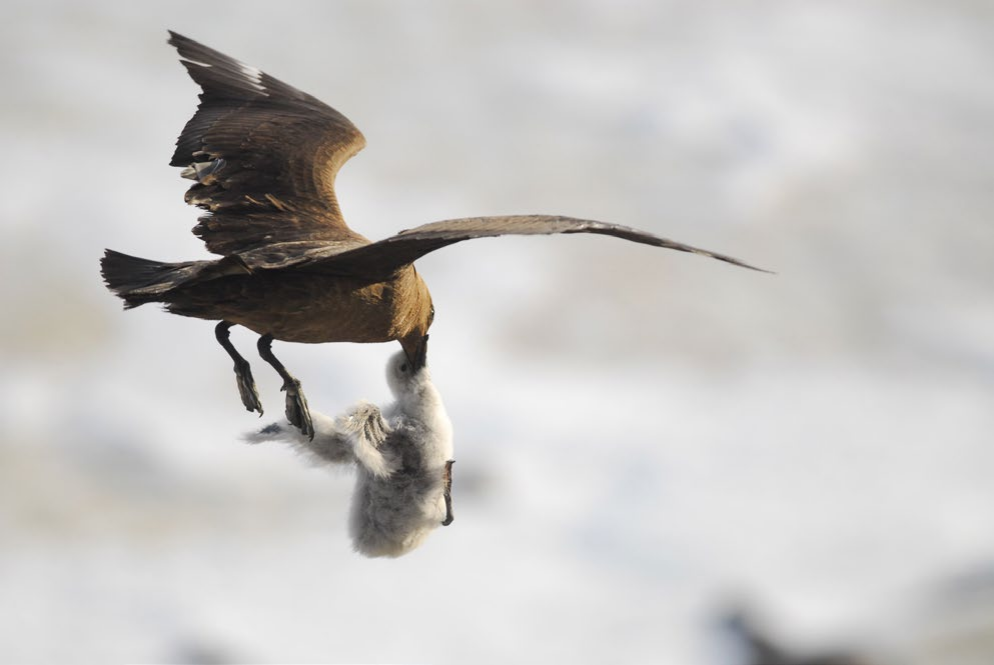
Organism photograph: south polar skua (*Catharacta maccormicki)* feeding on an Antarctic petrel (*Thalassoica antarctica*) chick. Credit: Sébastien Descamps

The Antarctic petrel is a medium‐sized seabird weighing approximately 600 g and is visible in Figure 1. It lays a single egg at the end of November/early December, and both parents contribute to incubation and chick rearing. Hatching occurs around mid‐January and fledging in early March (Lorentsen & Røv, 1995). The south polar skua weighs approximately 1,300 g and usually lays two eggs (Young, 1963b) between early December to late January and is again visible in Figure 1. Both parents incubate and feed the chicks, and during the breeding season (November–March), the south polar skuas at Svarthamaren feed almost exclusively on Antarctic petrel eggs and chicks (Brooke et al., 1999). Snow petrels are few in number at Svarthamaren, and breed in rock crevasses that are relatively inaccessible to south polar skuas, and as such are insignificant within the scope of this study. South polar skuas usually maintain exclusive feeding territories (Trillmich, 1978), but here seem to maintain only breeding territories while overlapping in terms of feeding. Additionally, this species is known to engage in intraspecific kleptoparasitism (Brooke et al., 1999), though the frequency of this at Svarthamaren and its effect on skua habitat use are unknown.

### 2.1 Skua tracking data

To understand skua foraging behavior, we tracked 47 individuals between 11 December 2012 and 17 February 2013 (10 individuals were tracked twice within this study, totaling 57 tracking periods). Sample size information is available in Table 1. Skuas were captured with a net attached to a 2‐meter pole, with a baited trap (triggered at a distance) or with an air‐propelled net gun, and handling time per skua was around 10 min. Each skua was fitted with an IGotU GPS logger taped around its tail feathers. Loggers weighed less 18 g (<2% of skua body mass) and recorded location data on average every 7.5 min (recording frequency interval was <20 min in 96.8% of cases, clustered heavily around the mean with *SD* = 35 s) for a period of around 11.1 ± 4.8 days (range: 3–24.9 days). Skua breeding status (incubation or chick rearing) was recorded at both logger deployment and retrieval, and all skua nests were monitored two to three times a week to estimate their hatching date. Skua nests were located on average 95.80 ± 68.83 m from the petrel colony.

**Table 1:** Sample sizes for skuas tracked at Svarthamaren, December 2012–February 2013

	Overall			Skua incubation		Skua chick rearing		Petrel incubation		Petrel chick rearing	
	All (*N* = 47)	M (*N* = 18)	F (*N* = 18)	M (*N* = 3)	F (*N* = 4)	M (*N* = 17)	F (*N* = 18)	M (*N* = 5)	F (*N* = 7)	M (*N* = 15)	F (*N* = 18)
Total time inside petrel colony (hr)	1,470.39	477.65	886.19	72.40	54.07	405.24	830.97	107.41	89.55	370.23	796.64
Total time outside petrel colony (hr)	27,347.22	4,385.85	19,192.63	697.39	1,187.43	3,688.46	17,863.41	1,315.54	2,355.39	3,070.31	16,837.23
Points inside petrel colony	8,081	4,829	2,567	431	321	4,398	2,239	641	531	4,188	2036
Points outside petrel colony	116,979	37,873	51,954	4,152	7,063	33,721	44,046	7,822	13,989	30,051	37,965
No. of tracks	57	20	24	3	4	18	22	5	7	16	18

Note

Sample sizes are shown for both sexes during periods of predator chick incubation and rearing, prey chick incubation and rearing, and throughout the tracking period.

Of the 47 individuals tracked, 36 were able to be sexed. Within these, we were able to track both members of a breeding pair in 11 cases (22 birds), and we tracked one member of the breeding pair in the other 14 cases. Most tracked skuas were sexed using either morphological measurements when both partners were known (i.e., females being larger than males in a given pair; Catry, Phillips, & Furness, 1999) or using genetic analyses where possible—DNA was extracted from blood sampled as part of another study on contaminant and isotopes, and primers M5 (Bantock, Prys‐Jones, & Lee, 2008) and P8 (Griffiths, Double, Orr, & Dawson, 1998) used to determine sex. Individuals of unknown sex were excluded from this study.

### 2.2 Antarctic petrel colony survey

**Figure 2:**
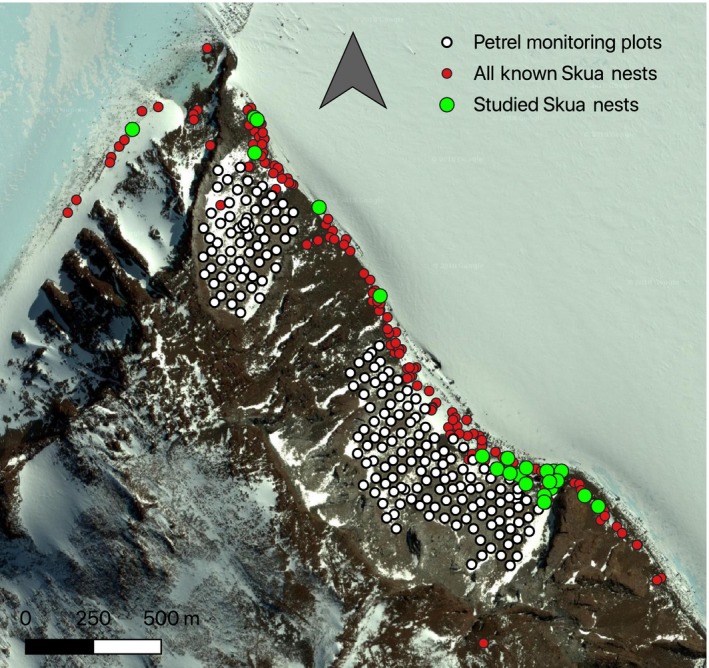
Locations of south polar skua nests, with nests used in this study highlighted in green. Petrel nest monitoring plot locations are also shown

**Figure 3:**
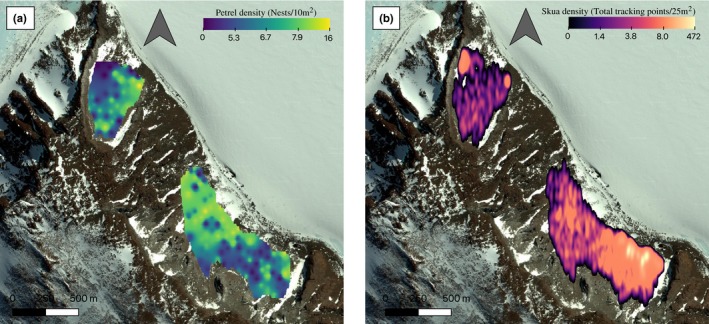
(a) Density of petrel (prey) nests in the study site. (b) Density of skua (predator) tracking points

To determine Antarctic petrel density, nests were surveyed using a grid of approximately 200 monitoring plot locations distributed at approximately 30 m intervals throughout the petrel colony, and the number of active nests (i.e., nests with an incubating bird) within an approximately 10 m^2^ area (1.78 m radius) of each monitoring point was recorded in early December (Figure 2, see Lorentsen, Røv, & Bangjord, 1993 for details on the protocol). These numbers were used to estimate petrel density in the whole colony at the beginning of the breeding season to understand whether prey density was a determinant of predator behavior and had not simply been reduced by predation activity throughout the season. We determined the outline of the colony using the *concaveman* function of the “concaveman” package, version 1.0.0 (Gombin, Vaidyanathan, & Agafonkin, 2017), to surround all active nests for the two regions of the colony separated by a rocky outcrop (Figure 3). Nest monitoring plots with zero active petrel nests at the colony edges were excluded to more accurately define the colony boundary.

In order to produce a continuous map of petrel nest density from the monitoring plot point data, nest density was interpolated across the study site (on a continuous scale) separately across the two colony regions using the *idw* function of the spatstat package (Baddeley, Rubak, & Turner, 2015). This was done separately in order to avoid inaccurate interpolation across the central area between the colony halves, where there are no petrel nests. Skuas can and do readily transit between the two petrel regions, and therefore, we then merged the rasters for the two petrel regions to produce a single layer for the whole colony. Skua tracking point data were then intersected with the petrel colony boundary polygon to exclude trips outside of the colony (i.e., nonfeeding trips, e.g., to bathing ponds), and values for petrel nest density extracted for each point in the skua telemetry data within this colony.

In addition to the whole colony survey, petrel nests were monitored every other day in four control plots (15 m × 15 m) to estimate the median hatching date in the colony (details in Descamps et al., 2016).

### 2.3 Data analyses

To understand whether Antarctic petrel density influenced south polar skua foraging behavior, we used resource selection functions (Manly, 2002) to compare petrel density throughout the colony (i.e., available habitat) to the petrel density where skuas were foraging (i.e., used habitat). Available habitat points were generated by selecting ten random points within the petrel colony (pseudoabsences) for every used tracking point (Northrup, Hooten, Anderson, & Wittemyer, 2013; Warton & Shepherd, 2010) using the *sampleRandom* tool in the raster package, version 2.8–4 (Hijmans et al., 2019), constrained by the colony boundary. This availability sample was then weighted so that used points were weighted ten times as heavily as their associated ten available points, to mitigate the disproportion in numbers of used points (known skua locations) versus available points, or pseudoabsences (Aarts, Fieberg, & Matthiopoulos, 2012). Petrel density for all used and available points was extracted from an inverse distance weight interpolation of the number of petrel nests per plot across the whole petrel colony, detailed above.

Resource selection functions were run as general linear models using the function *glmer* in the package “lme4”, version 1.1‐18‐1 (Bates, Maechler, & Bolker, 2013), using a binomial error distribution and logit link function. To test the influence of petrel density on skua habitat selection, we compared habitat use to petrel density, as a fixed effect. Habitat use was presented as a binary variable, where 1 = used area of habitat and 0 = pseudoabsences, a random sample of points drawn from the region of interest, and used as a surrogate when true absences are unknown (Hanberry, He, & Palik, 2012; Ward, Hastie, Barry, Elith, & Leathwick, 2009).

In addition to testing the effect of petrel density, we also tested the effects of skua and petrel breeding stage, and skua sex on habitat preference. To account for the potential effect of breeding stage on skua behavior, we fitted breeding stage of the tracked individual as a binary fixed effect: incubating or chick rearing as a factor. Similarly, to investigate whether skua foraging behavior changes depending on whether they are feeding primarily on eggs or chicks we fitted petrel breeding stage as a binary fixed effect using the population median hatch date.

To account for potential sex differences in skua foraging behavior, skua sex was fitted as a fixed effect. To investigate whether the propensity for skuas to forage in areas of high Antarctic petrel density changes when Antarctic petrel chicks are able to contribute to group vigilance and defense, we included a two‐way interaction between petrel density and petrel breeding stage. To investigate whether the propensity for skuas to forage in areas of high Antarctic petrel density changes according to whether they are provisioning chicks, we included a two‐way interaction between petrel density and skua breeding stage. Additionally, to determine whether there were sex differences in use of high Antarctic petrel density areas, we included a two‐way interaction between petrel density and skua sex. To account for multiple observations for both individuals and nests, skua ID and skua nest ID were fitted as random effects, with skua ID nested within nest ID.

We performed model selection with the Akaike Information Criterion adjusted for small samples (AICc). If the difference in AICc values between two models was <2, the models have equal statistical support, and in the case of nested models, the simplest was preferred (Burnham & Anderson, 2002). Model selection results are given in Table 2. Finally, post hoc comparisons were run using Tukey's tests, via *emmeans* version 1.4.1 (Lenth, 2019).

**Table 2:** Model selection results (dredge output), displaying the top twenty best fitting models found via the dredge function, ranked by AICc value, as well as the null model, single term models, and a model excluding interactions

Model rank	Model	Number of parameters	AICc	ΔAICc	Deviance
1	A_o_ + (SBS + PBS + Sex) × PD	8	194,285.50	0	194,265.50
2	A_o_ + (PBS + Sex) × PD + SBS	7	194,316.20	30.73	194,298.20
3	A_o_ + (PBS + Sex) × PD	6	194,319.60	34.15	194,303.60
4	A_o_ + (PBS + SBS) × PD	6	194,374.30	88.86	194,358.30
5	A_o_ + (PBS + SBS) × PD + Sex	7	194,375.80	90.27	194,357.80
6	A_o_ + PBS × PD + SBS	5	194,408.30	122.83	194,394.30
7	A_o_ + PBS × PD	4	194,409.50	124.06	194,397.50
8	A_o_ + PBS × PD + SBS + Sex	6	194,409.70	124.21	194,393.70
9	A_o_ + PBS × PD + Sex	5	194,410.90	125.37	194,396.90
10	A_o_ + (SBS + Sex) × PD + PBS	7	194,636.50	351.02	194,618.50
11	A_o_ + (SBS + Sex) × PD	6	194,641.60	356.16	194,625.60
12	A_o_ + SBS × PD + PBS	5	194,704.10	418.57	194,690.10
13	A_o_ + SBS × PD + PBS + Sex	6	194,705.20	419.76	194,689.20
14	A_o_ + SBS × PD	4	194,706.00	420.49	194,694.00
15	A_o_ + SBS × PD + Sex	5	194,707.20	421.70	194,693.20
16	A_o_ + Sex × PD + PBS	5	195,594.40	1,308.88	195,580.40
17	A_o_ + Sex × PD + PBS + SBS	6	195,595.20	1,309.70	195,579.20
18	A_o_ + Sex × PD	4	195,598.70	1,313.21	195,586.70
19	A_o_ + Sex × PD + SBS	5	195,599.50	1,314.04	195,585.50
20	A_o_ + PBS+PD + Sex	4	195,645.30	1,359.87	195,633.30
<20	A_o_ + PD + PBS + SBS + Sex	5	195,646.70	1,361.20	195,632.70
<20	A_o_	1	199,907.80	5,622.30	199,901.80
<20	A_o_ + PBS	2	199,909.30	5,623.80	199,901.30
<20	A_o_ + Sex	2	199,909.70	5,642.20	199,901.70
<20	A_o_ + SBS	2	199,909.70	5,642.20	199,901.70

Note

A_o_ = null model, SBS = skua breeding stage, PBS = petrel breeding stage, PD = petrel density, and Sex = Skua sex.

All analyses were performed with software R version 3.5.0 (R Development Core Team, 2016).

## 3 RESULTS

On average, skuas spent 5.1% ± 2.1 (mean ± *SE*) of their time in the petrel colony (Table 3) and females spent less time within the colony than males (4.4% ± 0.01 *SE* vs. 9.8% ± 3.0 *SE*). Petrel density varied within the colony from 0 to 16 nests per 10 m^2^ sample plot, while the total number of skua tracking points per 25 m^2^ area ranged between 0 and 472 (Figure 3).

**Table 3:** Mean time spent both inside and outside the petrel colony by south polar skuas tracked at Svarthamaren, December 2012–February 2013

	Overall			Skua incubation		Skua chick rearing		Petrel incubation		Petrel chick rearing	
	All	M	F	M	F	M	F	M	F	M	F
Mean time in colony (hr)	25.80 ± 10.73 (5.10)	23.88 ± 6.96 (9.82)	36.92 ± 24.88 (4.41)	24.13 ± 13.26 (9.40)	13.51 ± 6.42 (4.35)	22.51 ± 7.37 (9.90)	37.77 ± 27.19 (4.44)	21.48 ± 10.42 (7.55)	12.79 ± 4.91 (3.66)	23.14 ± 8.35 (10.76)	44.26 ± 33.19 (4.52)
Mean time out of colony (hr)	479.78 ± 228.28 (94.90)	219.29 ± 21.43 (90.18)	799.69 ± 541.21 (95.19)	232.46 ± 38.52 (90.60)	296.86 ± 43.82 (95.65)	204.91 ± 24.07 (90.10)	811.97 ± 591.90 (95.56)	263.11 ± 34.55 (92.45)	336.48 ± 53.07 (96.34)	191.89 ± 21.76 (89.24)	935.40 ± 723.76 (95.48)

Note

Values are averages (in hr) ± *SE*, with percentages in parentheses. M represents males and F females.

**Figure 4:**
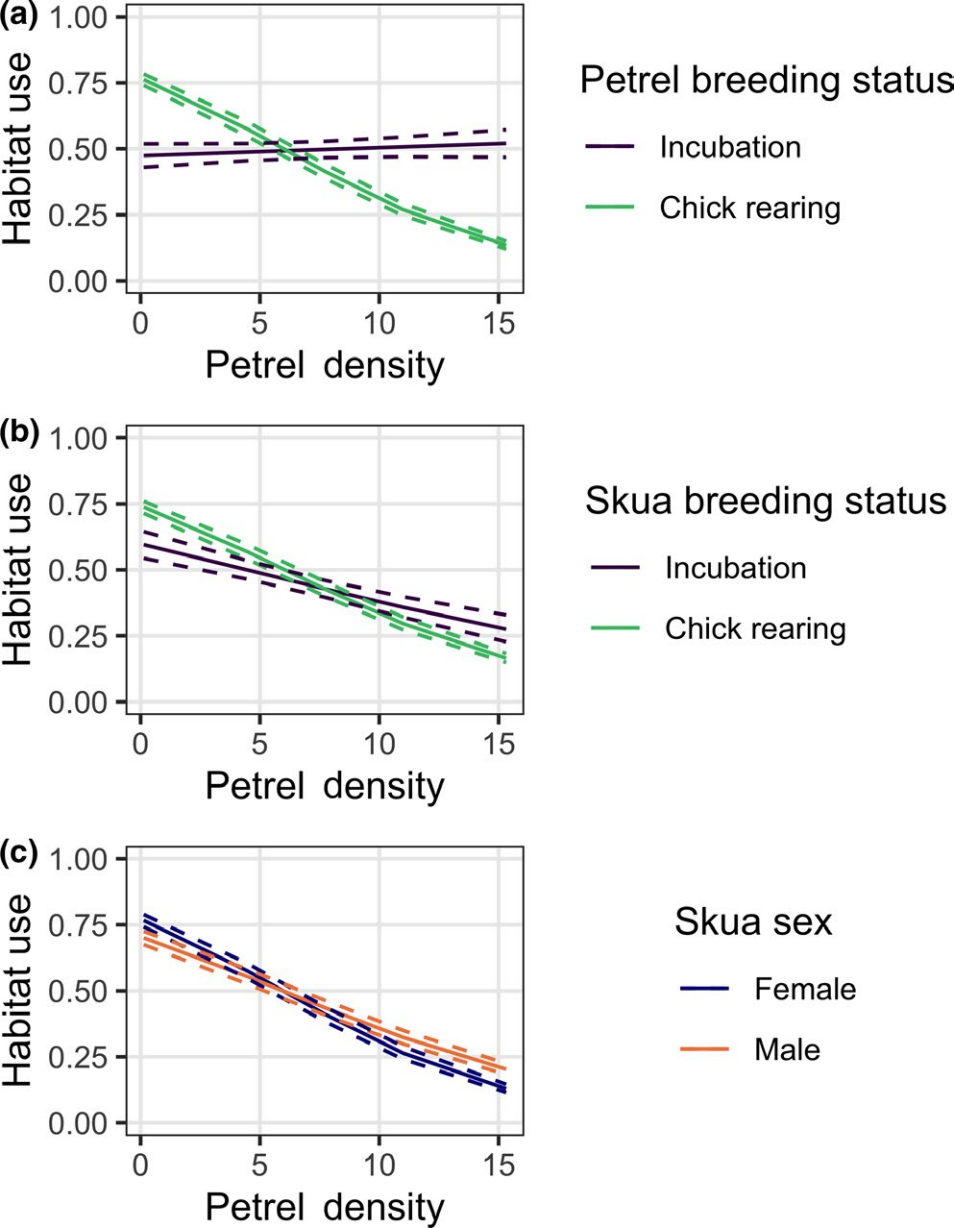
Probability of skua using a particular habitat at a given petrel density level, (number of petrel nests per 10 m^2^ sample plot), as an interaction with (a) petrel breeding status, (b) skua breeding status, and (c) skua sex

Petrel density significantly affected the likelihood of a skua foraging in a particular area, with areas of higher petrel density being on average less likely to be used by skuas (Table 4, parameter estimate ± *SE *= −0.12 ± 0.06). This pattern is visible in Figure 3, where high skua density areas predominantly coincided with areas of low petrel density. However, this pattern is dependent on both the petrel and skua breeding status and is also a function of the skua sex (Table 4). During petrel incubation, skuas showed no preference for any particular prey densities (Figure 4a, parameter estimate ± *SE* = −0.02 ± 0.06). In contrast, during petrel chick rearing, skuas exhibited a strong preference for areas of lower prey density (Figure 4a, −0.1 ± 0.06; Table 4). When incubating eggs, skuas showed a stronger selection for areas of low petrel density than when rearing chicks (Figure 4b, −0.2 ± 0.07 and −0.1 ± 0.06, respectively; Table 4). Female skuas showed a slightly stronger preference for areas of low petrel density compared with males (Figure 4c, −0.1 ± 0.06 and −0.1 ± 0.06, respectively; Table 4).

**Table 4:** South polar skua habitat selection according to Antarctic petrel density, sex, colony mean petrel breeding status, and individual skua breeding status

Coefficient	Level	Petrel density selection	Parameter estimate	Post Hoc comparison
Model intercept	**Whole population**	**Negative**	**−0.12 ± 0.06**	**NA**
Petrel breeding status	Incubating	Not‐significant	−0.02 ± 0.06	*p* < .05
	**Chick rearing**	**Negative**	**−0.13 ± 0.06**	
Skua breeding status	**Incubating**	**Negative**	**−0.20 ± 0.07**	*p* < .05
	**Chick rearing**	**Negative**	**−0.11 ± 0.06**	
Sex	**Female**	**Negative**	**−0.14 ± 0.06**	*p* = .19
	**Male**	**Negative**	**−0.11 ± 0.06**	
Petrel Density	**NA**	**Positive**	**0.11 ± 0.02**	**NA**
Petrel Breeding Status	**NA**	**Negative**	**−0.11 ± 0.03**	**NA**
Skua Breeding Status	**NA**	**Positive**	**0.09 ± 0.04**	**NA**
Sex	NA	Not‐significant	0.03 ± 0.03	NA

Note

Parameter estimates are given from the full model of skua habitat selection, averaged for mean petrel density in the case of factorial variables (sex and breeding status). Bold type indicates significant habitat selection by petrel density. Post hoc comparisons were run using the Tukey tests, with results given in column five.

Post hoc Tukey's testing indicated statistically significant differences in skua prey selection between incubating and chick rearing stages in both petrels and skuas (*p* < .05), and no significant difference between skua sexes (*p* = .19), with results shown in Table 4.

## 4 DISCUSSION

Our study demonstrates a clear association between south polar skua habitat use and the density of their sole prey species during the breeding season, Antarctic petrels. We observed that during petrel incubation, skua habitat use was not affected by petrel density; however, while petrels were rearing chicks, skuas avoided areas of high petrel density. The responses shown here are not necessarily linear in nature, however.

Previous studies have found contrasting relationships between the density of prey species and predator foraging behavior (Brunton, 1997; Reitsma, 2008; Schmidt & Whelan, 1999; Stokes & Boersma, 2000). Stokes and Boersma (2000), for example, described a positive correlation between predation and prey density in Magellanic penguins (*Spheniscus magellanicus*). They found no evidence for group defense or antipredation benefits at high densities in this species and suggested that increased intraspecific aggression in higher densities would attract predators. In contrast, Becker (1995) recorded that common tern (*Sterna hirundo*) chicks in higher density clusters experienced the lowest predation and highest survival rates, due to an increased group defense effect correlated with an increased number of adult terns present to attack predators.

Here, we found that while adult petrels were defending nests and incubating eggs, skuas exhibited no preference for areas of differing prey density, suggesting an optimal foraging balance between maximizing access to prey and minimizing risk from prey defense (Sih, 1984). The lack of preference for foraging among particular petrel densities during the petrel incubation phase may emerge if adult petrels can adequately defend their nests alone, and so neighborhood density does not affect predation rates. However, when chicks have hatched and are left unguarded or guarded by just one parent (Varpe, Tveraa, & Folstad, 2004), they replace the missing parent's contribution to group vigilance and predator defense, namely by spitting oil as a defensive tactic (Warham, 1977), and as a result skuas strongly avoid areas of high prey density after petrel hatching. It is important to note, however, that the high density of nests could prevent skuas from landing, and it is difficult to disentangle this effect from the effects of group vigilance in reducing predation in high density areas. This demonstrates the importance of antipredator tactics in determining predator behavior and can explain the disparity of the prey density effect among colonial breeding seabirds that successfully employ group antipredator defense (e.g., petrels here and terns, mentioned above) compared to those that do not.

Skua foraging habits are therefore likely influenced by the ability of petrel chicks to contribute to antipredator defense; the effectiveness of which is enhanced in areas of high petrel density, presumably because of improved group vigilance. This conclusion is based on skua habitat selection, which we assume to be correlated with foraging activity, as skuas spent very little time in the petrel colony (on average 5.1% of tracking duration, ±2.1%), suggesting that they use the colony for feeding only and that time investment in territory defense is important within this system.

Denser aggregations or larger group sizes have been shown to yield fitness benefits to individuals because of lower predation rates in a range of systems including bank swallows, *Riparia riparia* (Hoogland & Sherman, 1976), prairie dogs, *Cynomys leucurus* and *C. ludovicianus* (Hoogland, 1981), Montagu's harriers, *Circus pygargus* (Arroyo, Mougeot, & Bretagnolle, 2001), and other colonial seabirds such as common guillemots, *Uria aalge* (Birkhead, 1977). In accordance with these studies, our results show that in our study system, predators are less likely to utilize habitats with higher prey densities, reducing predation in these areas. Foraging in areas of high petrel density may indicate risk‐taking behavior, given the propensity of petrels to spit oil that is harmful to skua plumage.

Propensity to take risk may increase with progression of a breeding attempt, as individuals become increasingly invested and so have more to lose (Forbes, Weatherhead, & Armstrong, 1994; Magnhagen & Vestergaard, 1991). Boukhriss and Selmi (2010) observed this behavior in rufous bush robins (*Cercotrichas galactotes*), in which distance of retreat after nest flushing decreased with incubation stage and progress through the breeding season, as nest value increased and opportunities for renesting dwindled. However, we observed that skuas were more averse to foraging within high petrel density areas during their own chick rearing stage compared with during incubation. This suggests that the propensity of skuas to take risk may in fact decrease with greater investment in an individual reproductive attempt, perhaps because later in the breeding season individuals prioritize their own survival (Halupka & Halupka, 1996).

Both male and female skuas preferred to forage in areas of lower petrel density; however, males were slightly less selective and were more likely to forage in high petrel density areas than females. Furthermore, males spent more time inside the petrel colony than females. These results could therefore support the hypothesis that reversed size dimorphism in skuas (i.e., females are larger than males) may help facilitate females relegating their partner to the role of food provider (Catry et al., 1999; Smith, 1982). This is however speculative at this stage and would need further investigation.

## 5 CONCLUSION

Our study provides a rare opportunity to assess the relationship between predators and prey in a system without potentially confounding factors such as alternative prey sources or multiple predatory species. We found that south polar skuas preferentially foraged in areas of low petrel density during petrel chick rearing. These findings may have important implications for prey population dynamics. After an initial decrease in density (due to predation or lower breeding propensity), localized petrel populations could well continue to decline further due to the loss of group defense effectiveness, increasing attractiveness to predators. This will then lead to a negative feedback loop and nonlinear decline in parts of the colony. Our study opens a path for future research aiming to quantify the impact of predation on petrel population dynamics and to assess the numerical and functional response of skua with changes in petrel numbers.

## CONFLICT OF INTEREST

The authors declare no competing interests.

## AUTHOR CONTRIBUTIONS

SD designed the study. KB, AT, and SP analyzed the data. All authors contributed to writing the paper.

## Data Availability

Data used in this study is freely available on the Dryad data repository (datadryad.org) at https://doi.org/10.5061/dryad.3tx95x6bp
